# Dietary and related data collected during pregnancy in the Avon Longitudinal Study of Parents and Children (ALSPAC)

**DOI:** 10.12688/wellcomeopenres.23464.2

**Published:** 2025-02-24

**Authors:** Caroline M Taylor, Kate Northstone, Jean Golding, Louise Jones, Genevieve Buckland, Pauline M Emmett

**Affiliations:** 1 Centre for Academic Child Health, Canynge Hall, University of Bristol Medical School, Bristol, England, UK; 2 Population Health Sciences, University of Bristol Medical School, Bristol, England, UK; 3 Centre for Public Health, University of Bristol Medical School, Bristol, England, UK

**Keywords:** ALSPAC, Pregnancy, Diet, Nutrition, Woman, Dietary patterns

## Abstract

The Avon Longitudinal Study of Parents and Children (ALSPAC) is a longitudinal birth cohort study based in the south-west of the UK. Pregnant women resident in and around the city of Bristol with expected delivery dates between 1 April 1991 and 31 December 1992 were invited to take part. The initial number of pregnancies enrolled was 14,541. Four questionnaires were sent to women through the post during pregnancy (including a food frequency questionnaire (FFQ) at 32 weeks) for self-completion; excluding withdrawals and exclusions, 11,978 FFQ records are available (April 2024). The main part of the FFQ at 32 weeks comprised questions on the weekly frequency of consumption of 43 food groups and food items. More detailed questions covered a further eight foods/drinks normally consumed daily. The data were used to derive daily nutrient intakes for each participant. Dietary patterns were derived using principal components analysis. Together with the diverse ALSPAC resource containing detailed data on demographics, lifestyle, environment, genetics and health, these data form a unique resource for the study of: (1) maternal diet in pregnancy, fetal/child development and their life course; (2) women’s diet and their life course.

## Introduction

As previously documented ^1, 2^, prospective studies such as the Avon Longitudinal Study of Parents and Children (ALSPAC) have significant methodological advantages for investigation of the associations between exposures and outcomes. Dietary intakes are some of the most important exposures for studying health and development. However, it is essential that researchers have a full understanding of the methods of collection and processing of dietary data in observational studies in order to ensure that the methods are appropriate for their research question and to enable correct interpretation of data analysis. Here we describe the dietary data available from pregnant women enrolled in ALSPAC, a longitudinal observational cohort study in the UK. We describe how the dietary data were collected and processed, and the derived variables that are currently available to researchers. We also describe the processing and classification of data to provide further derived diet-related variables if required.

## Methods

### Summary

Pregnant women (known as Generation 0: G0) resident in Avon, UK with expected dates of delivery between 1st April 1991 and 31st December 1992 were invited to take part in the ALSPAC study. 20,248 pregnancies were identified as eligible and the initial number of pregnancies enrolled was 14,541 (71.8% of those eligible). Of the initial pregnancies, there was a total of 14,676 fetuses, resulting in 14,062 live births; of the original 14,541 initial pregnancies, 338 were from women who had already enrolled with a previous pregnancy, meaning 14,203 unique mothers were initially enrolled in the study ^3– 5^.

The offspring (G1) were born in 1991/2 and are now being followed as adults, so the data provide an invaluable resource to assess the contribution of pregnancy diet to growth and developmental outcomes of the offspring in childhood and adulthood, as well as the life course of women. The dietary data are enriched by the availability of a range of variables to act as confounders, as well as health and developmental outcomes in the mother and child.

There are data from 11,978 food frequency (FFQ) records and from other diet-related variables available to researchers (April 2024). The median gestational age at completion of the FFQ was 32 weeks’ gestation (IQR 32, 33). The demographics of the participants are shown in Table 1.

**Table 1.** Demographics of ALSPAC participants: core cohort at 32 weeks’ gestation and those who completed the food frequency questionnaire (FFQ) at 32 weeks’ gestation.

	Core cohort (max. n=14,569)	FFQ completers (max. n=11,978)
Education		
O level or below	7968 (64.7%)	7688 (64.6%)
A level or degree	4344 (35.3%)	4220 (35.4%)
Age at completion of Questionnaire C (years)	28.6 4.9 ± (n=11,896)	28.6 4.8 ± (n=11,827)
Housing tenure		
Rented/Other	3577 (26.8%)	2819 (24.3)
Mortgaged/Owned	9731 (73.2%)	8530 (75.7%)
Ethnicity		
White	11,901 (97.4%)	11526 (97.4%)
Other than white	320 (2.6%)	302 (2.6%)
Smoking at completion of Questionnaire C		
Yes	2410 (20.0%)	2377 (19.8%)
No	9647 (80.0%)	9601 (80.2%)
Pre-pregnancy BMI (kg/m ^2^)	23.0 ± 3.8 (n=11,496)	22.9 ± 3.8 (n=10,602)

Highest education level achieved: O level, Ordinary level certificate (typically at 16 years); A level, Advanced level certificate (typically at 18 years)

The G0 data have been used in a variety of categorisations for analyses including single food items (e.g. fish/seafood ^6^), single nutrients (e.g. vitamin B _12_^7, 8^, *n*-3 fatty acids ^9^), macronutrients ^10^, dietary styles (e.g. vegetarian ^11^), food groups (e.g. fruits/vegetables ^12^, meat ^13^, milk and dairy products ^14^), dietary variety ^15^, diet quality scores ^16, 17^ and dietary patterns ^18, 19^ to model associations with a range of health outcomes in the mother (e.g. exposure to toxins ^18, 20, 21^, eating disorders ^22^) and health and development outcomes in the child (e.g. visual development ^23^, behaviour and IQ ^24– 26^, blood pressure ^27^, hepatic steatosis ^28^, bone mass ^17^, speech development and mathematical abilities ^8^, smoking behaviours ^6^, hypospadias ^11^) (see Table 2).

**Table 2.** Examples of ALSPAC pregnancy diet data used in research studies.

Type of dietary variable	Diet variable in pregnancy	Author	Outcome group		Outcome
			Group	Age/timing	
**Nutrients**	Multiple nutrients	Leary *et al.* ^27^	Child	7 years	Blood pressure
	Multiple nutrients	Rogers *et al.* ^29^	Mother/Child	During pregnancy/Birth	Financial difficulties, smoking habits and birthweight
	Macronutrients	Brion *et al.* ^10^	Child	9–11 years	Diet and body composition
	Calcium, iron	Taylor *et al.* ^30^	Mother	During pregnancy	Lead exposure
	Vitamin B _12_	Bonilla *et al.* ^7^	Child	8 years	IQ
	Vitamin B _12_	Golding *et al.* ^8^	Child	24, 38 months; 6, 8–9, 10–11, 13 years	Speech and language development/ mathematical abilities
	*n*-3 Fatty acids	Waylen *et al.* ^9^	Child	3 years	Externalising behaviour
	*n*-3 Fatty acids	Golding *et al.* ^31^	Mother	During pregnancy	Depressive symptoms
	*n*-3 Fatty acids	Rogers *et al.* ^32^	Child	Birth	Intrauterine growth retardation
**Food/food groups**	Fish/seafood	Daniels *et al.* ^33^	Child	15–18 months	Cognitive development
	Fish/seafood	Hibbeln *et al.* ^24^	Child	6 months to 8 years	Neurodevelopment scores including IQ
	Fish/seafood	Gow *et al.* ^6^	Mother	During pregnancy	Smoking patterns
	Fish/seafood	Golding *et al.* ^25^	Mother	During pregnancy	Mercury exposure
	Fish/seafood	Hibbeln *et al.* ^34^	Child	7–13 years	Scholastic ability
	Fish/seafood	Golding *et al.* ^25^	Child	8 years	IQ
	Fish/seafood	Golding *et al.* ^35^	Child		Cognitive development (review)
	Fish/seafood	Gregory *et al.* ^36^	Child	7–17 years	Blood pressure
	Fish/seafood	Taylor *et al.* ^37^	Child	Birth	Birth outcomes
	Fish and processed foods	Mesirow *et al.* ^38^	Child	4–13 years	Early onset persistent conduct problems
	Oily fish	Williams *et al.* ^23^	Child	3.5 years	Stereoacuity
	Fruits/vegetables	de Lauzon-Guillain *et al.* ^12^	Child	2–4 years	Early feeding practices/fruit and vegetable intakes
	Meat	Hibbeln *et al.* ^13^	Child	13 years	Substance misuse
	Milk and dairy products	Dineva *et al.* ^14^	Mother	During pregnancy	Iodine status
	Free sugars	Sekkarie *et al.* ^28^	Mother	24 years postpartum	Hepatic steatosis
**Dietary patterns**	PCA, CA, RRR	Emmett *et al.* ^39^	Child		Child’s dietary pattern, nutrient profiles
	PCA	Northstone *et al.* ^19^	Mother	During pregnancy	Nutrient intakes
	PCA	Northstone *et al.* ^40^	Mother	During pregnancy	Sociodemographic and lifestyle factors
	PCA	Northstone *et al.* ^41^	Mother	During pregnancy	Adjustment for energy intakes
	PCA	Taylor *et al.* ^18^	Mother	During pregnancy	Lead exposure
	PCA	Taylor *et al.* ^21^	Mother	During pregnancy	Cadmium exposure
	PCA	Micali *et al.* ^22^	Mother	Lifetime	Eating disorders
	PCA	Pervin *et al.* ^42^	Mother	12 years postpartum	Dietary pattern trajectories
	PCA	Coathup *et al.* ^43^	Mother	During pregnancy	Alcohol consumption
	PCA	Molyneaux *et al.* ^44^	Mother	During pregnancy	Depression, gestational weight gain
	PCA	Shaheen *et al.* ^45^	Child	7 year, 8–9 years	Asthma
	PCA plus *n*-3 fatty acids from fish	Vaz *et al.* ^46^	Mother	During pregnancy	Anxiety
	CA	Freitas-Vilela *et al.* ^26^	Child	8 years	IQ
	CA	Freitas-Vilela *et al.* ^47^	Child	7 years	Nutrient intakes and dietary patterns
	RRR	Marks *et al.* ^20^	Mother	During pregnancy	Exposure to persistent endocrine-disrupting chemicals
	CFA/LCA	Pina-Camacho *et al.* ^48^	Mother	Child	Dysregulation
**Other**					
Dietary style	Vegetarian	North & Golding ^11^	Child	Birth	Hypospadias
Dietary variety scores	Healthy plate variety score	Jones *et al.* ^49^	Child	2–4 years	Healthy plate variety score
Diet quality scores	Dietary Inflammatory Index	Woolford *et al.* ^17^	Child	9 years	Bone mass
	Dietary Approaches to Stop Hypertension (DASH) and Energy-adjusted Dietary Inflammatory Index (E-DII)	Aubert *et al.* ^16^	Mother	During pregnancy	Diet quality and anti-inflammatory diet
	Dietary Approaches to Stop Hypertension (DASH) and Energy-adjusted Dietary Inflammatory Index (E-DII)	Chen *et al.* ^50^	Child	Birth	Birth outcomes
	Dietary Approaches to Stop Hypertension (DASH) and Energy-adjusted Dietary Inflammatory Index (E-DII)	Mensink-Bout *et al.* ^51^	Child		Respiratory outcomes

CA, cluster analysis; CFA, confirmatory factor analysis; LCA, latent class analysis; PCA, principal components analysis; RRR, reduced rank regression. For author-derived variables not available in the ALSPAC database, contact the corresponding author of the specific publication. See also Table 1 in Emmett *et al.* ^2^

### Ethics approval

Ethics approval for the study was obtained from the ALSPAC Law and Ethics committee and local research ethics committees. Implied consent for the use of data collected via questionnaires and informed consent for information acquired for tests in clinics were obtained from participants following the recommendations of the ALSPAC Ethics and Law Committee at the time ^52^ (Bristol and Weston Health Authority: E1808 Children of the Nineties: Avon Longitudinal Study of Pregnancy and Childhood (ALSPAC) (28 November 1989); Southmead Health Authority: 49/89 Children of the Nineties – ‘ALSPAC’ (5 April 1990); Frenchay Health Authority: 90/8 Children of the Nineties (28 June 1990)). Study participants have the right to withdraw their consent for elements of the study or from the study entirely at any time. Full details for the ALSPAC consent procedures are available on the study website ^53^. the conditions at the time of the questionnaires in pregnancy (1991–2) followed the principle of implied consent by return of the completed questionnaire, as stated in the data note, in line with ethics approval at that time. The website contains current information on consent for ongoing data collection in ALSPAC, in which informed written consent is required for questionnaires.

### Methods

***Collection of dietary variables in pregnancy (G0) in the ALSPAC database.*** All diet-related data were collected by three self-completed postal questionnaires. The complete questionnaires used by ALSPAC are available at: https://www.bristol.ac.uk/alspac/researchers/our-data/questionnaires/carer-questionnaires/.

Data collections related to dietary intake during pregnancy comprised:

- **Questionnaire A: At enrolment if <14 weeks’ gestation**
  Questionnaire A contained data collection on: (1) **non-alcoholic drinks** (tea, coffee, cola, milk, other); and (2) **alcoho**l (beer/lager, wine, spirits, other alcohol).
- **Questionnaire B: 18 weeks’ gestation**
  Questionnaire B contained data collection on: (1) **non-alcoholic drinks** (tea, coffee, cola, milk, other); (2) **alcohol** (beer, wine, spirits); (3) **supplements** (iron, zinc, calcium, folic acid, vitamins, other supplements or diet foods); (4) **food-related behaviours**.
- **Questionnaire C: 32 weeks’ gestation**
  Questionnaire C collected data on: (1) **foods/food groups** using the ALSPAC FFQ; (2) **other** additional questions on foods and drinks; (3) **drinks** (tea, coffee, cola, herbal tea); (4) **alcohol** (beer, wine, spirits, other alcohol, total alcohol units per week); (5) **supplements** (iron, zinc, calcium, folic acid, vitamins, other supplements or diet foods in past 3 months); (6) **food-related behaviours**.

Information concerning the timing of these questionnaires is available in Iles-Caven *et al.* ^54^. For a summary of diet-related variables and their timings collected in pregnancy in the ALSPAC database see Table 3. The ALSPAC FFQ used in pregnancy (included in Questionnaire C at 32 weeks’ gestation) was an unquantified FFQ aiming to cover all the main foods consumed in Britain at the time ^2^. The foods chosen for the FFQ were based on those used in a study in South Wales ^55^, and modified in the light of weighed food intake study in adults carried out in Avon ^56^.

**Table 3.** Summary of diet-related variables in pregnancy available in the ALSPAC database.

	Questionnaire A	Questionnaire B	Questionnaire C
	<14 weeks	18 weeks	32 weeks
Foods			
Foods/food groups	-	-	Yes
Other foods	-	-	Yes ^a^
Dietary supplements	-	Yes ^b^	Yes ^c^
Drinks			
Alcohol	Yes	Yes	Yes
Other drinks	Yes ^d^	Yes ^d^	Yes ^e^
Diet-related behaviours ^f^	-	Yes	Yes
Derived variables			
Nutrients	-	-	Yes
Dietary patterns	-	-	Yes

^a^Includes questions on: Types of bread; Take away meals per month; Types of fat for cooking and spreading; Types of milk used/milk use in drinks and foods, e.g. cereal; Sugar in tea and coffee; Herbal tea; Purchase of organic fruit, vegetables, meat, other, health foods; Vegetarian or vegan (yes/no, years) ^b^Iron, zinc, calcium, folic acid, vitamins, other supplements or diet foods during this pregnancy ^c^Iron, zinc, calcium, folic acid, vitamins, other supplements in past 3 months ^d^Tea and coffee (cups per day) (weekday, weekend, week; caffeinated, decaffeinated); Cola (number of cans) (weekday, weekend, week; caffeinated, decaffeinated); Milk (glasses per day, weekday, weekend, week): other drinks (weekday, weekend); Other drinks (number) ^e^Tea and coffee (cups per day) (caffeinated, decaffeinated); cola (number of cans per day) (caffeinated, decaffeinated) ^f^Includes questions on attitudes to body shape, concern over weight gain, etc. (see Table 5)

The FFQ included questions on the weekly frequency of consumption of 43 food groups and food items. Respondents were asked to tick one of the following options: Never or rarely/Once in 2 weeks/1–3 times per week/4–7 times per week/More than once a day.

Further detailed questions were asked about daily consumption and types of a further eight basic foods (bread, coffee, tea, sugar, milk, spreading fats, cooking fats, soft drinks), and about the ways in which food was prepared and eaten, for example whether some or all of the fat was cut off meat, how often food was fried and how many slices of bread (or similar) eaten in a day were spread with fat.

Dietary data collected in pregnancy including that collected by food frequency questionnaire (FFQ) in the ALSPAC database comprise: (1) consumption frequencies of foods/food groups ( Table 4); (2) additional variables (types of bread, fats and milk; use of sugar in beverages; consumption of soft drinks, tea/coffee and alcohol; use of takeout foods; purchase of organic foods; and being a vegetarian or vegan); (3) use of supplements; (4) food-related behaviours ( Table 5). All three questionnaires also included questions on alcohol consumption.

**Table 4.** List of all food groups and additional questions in G0 ALSPAC FFQ (Questionnaire C (32 weeks)).

Question number	Variable	Question	Response options
		**How many times a week nowadays do you eat:**	Never or rarely/Once in 2 weeks/1–3 times a week/ 4–7 times a week/More than once a day
C1a–r	c200	Sausages, burgers	〃
	c201	Pies, pasties (pork pie, steak/meat pie, etc.)	〃
	c202	Meat (beef, lamb, pork, ham, bacon, etc.)	〃
	c203	Poultry (chicken, turkey, etc.)	〃
	c204	Liver, liver pate, kidney, heart	〃
	c205	White fish (cod, haddock, plaice, fish fingers, etc.)	〃
	c206	Other fish (pilchards, sardines, mackerel, tuna, herring, kippers, trout, salmon, etc.)	〃
	c207	Shellfish (prawns, crab, cockles, mussels, etc.)	〃
	c208	Eggs, quiche	〃
	c209	Cheese	〃
	c210	Pizza	〃
	c211	Chips	〃
	c215	Roast potatoes (cooked in fat)	〃
	c216	Boiled mashed, jacket potatoes	〃
	c217	Rice (boiled)	〃
	c218	Pasta (e.g. spaghetti, pot noodles, lasagna)	〃
	c219	Crisps	〃
	c220	Fried foods (e.g. fried fish, eggs, bacon, chops)	〃
C3a–y	c222	Baked beans	〃
	c223	Peas, sweetcorn, broad beans	〃
	c224	Cabbage, Brussel sprouts, kale and other green leafy vegetables	〃
	c225	Other green vegetables (cauliflower, runner beans, leeks, etc.)	
	c226	Carrots	〃
	c227	Other root vegetables (turnip, swede, parsnip, etc.)	〃
	c228	Salad (lettuce, tomato, cucumber, etc.)	〃
	c229	Fresh fruit (apple, pear, banana, orange, bunch of grapes, etc.)	〃
	c230	Tinned juice (including tomato juice)	〃
	c231	Pure juice not in tin	〃
	c232	Pudding (e.g. fruit pie, crumble, cheesecake, milk pudding, mousse, gateaux)	〃
	c233	Oat cereals (e.g. porridge, Ready Brek, muesli)	〃
	c234	Wholegrain or bran cereals (e.g. All-Bran, Bran Flakes, Weetabix, Wheatflakes, Fruit & Fibre)	〃
	c235	Other cereals (e.g. Corn Flakes, Rice Krispies, Special K, Frosties)	〃
	c236	Cakes or buns (fruit cake, sponge, teacake, buns, doughnut, flapjack, scone, custard tart, cream cake, etc.)	〃
	c237	Crispbreads (Ryvita, crackerbread, etc.)	〃
	c238	Biscuits (digestive, shortcake, Hob Nobs, Rich Tea, Nice, Marie, chocolate biscuits, Penguin, Club, KitKat, etc.)	〃
	c239	Chocolate bars (Mars, Twix, Wispa, Bounty, Crème Egg, etc.)	〃
	c240	Pulses – dried peas, beans, lentils, chickpeas, etc.	〃
	c241	Nuts, nut roast	〃
	c242	Bean curd (e.g. tofu, miso)	〃
	c243	Tahini	〃
	c244	Soya ‘meat’, TVP, vegeburgers	〃
	c245	Chocolate (dairy milk or plain, nut, fruit-filled, etc.)	〃
	c246	Sweets (peppermints, boiled sweets, toffees, etc.)	〃
		**Additional questions relevant to the FFQ**	
C2	c221	Do you eat all the fat on meat?	Yes, all of it/Yes, Some of it/No/Never eat meat
C4	c247	When you have a soft drink, how often do you choose low calorie or diet drinks?	Always/Sometimes/Not at all/Don’t drink soft drinks
C5	c250	How many pieces of bread, rolls or chapatis do you eat on a usual day?	Less than 1/1–2/3–4/5 or more
C6	c251	How many times in a month do you eat take-away foods for your main meal?	Never or rarely/1–2/3–4/5–9/10 or more
C7a-e	c252–c256	What type of bread do you eat most days?	White bread/Brown or granary bread/Wholemeal bread/Chapattis or naan bread/Don’t usually eat any bread (Yes/No)
C8(i)–(ii)	c260–c274	What type of fat do you mainly use: Butter, ghee, dripping lard, solid cooking fat Hard or soft margarine e.g. Flora, sunflower, Vitalite Low fat spread e.g. Outline, Delight, St Ivel Gold Sunflower, soya, corn, olive oil Other vegetable oil Other	On bread or vegetables: Yes/No For frying: Yes/No [text] ^a^
C9	c275	How many slices of bread (or rolls) spread with fat do you eat each day (include bought sandwiches)	[number]
C10a–h	c276–c283	What types of milk do you use: Full fat (silver or gold top) Semi-skimmed (red stripe) Skimmed (blue stripe) Sterilised Dried milk Goat/sheep milk Soya milk Other (please describe)	Yes usually/Yes sometimes/No not at all [text] ^a^
C11a–f	c284–c289	How often do you have milk: In tea In coffee On breakfast cereal As pudding (custard, rice) To drink on its own As a milky drink (Horlicks, cocoa, all milk coffee)	Yes usually/Yes sometimes/No not at all
C12b,e	c302, c306	[Included in questions about tea/coffee drinking] How many spoons of sugar in each cup [of tea]? How many spoons of sugar in each cup [of coffee]?	[number] [number]

^a^Text available on request

**Table 5.** List of all food behaviour questions in G0 ALSPAC questionnaires.

Questionnaire	Variables	Questions	Response options
**B (18 weeks)**	b800–b820	Have you ever actually made yourself sick (vomit) because you wanted to lose weight or because you had eaten too much?	Yes/No
		If yes, how often have you made yourself vomit during this pregnancy?	Not at all/Once/2–4 times/5–14 times/15 or more times
		Have you ever taken laxatives because you wanted to lose weight or because you had eaten too much?	Yes/No
		If yes, how often have you done so during this pregnancy?	Not at all/Once/2–4 times/5–14 times/15 or more times
		Over the past four weeks (28 days) ^a^:	
		Has thinking about your shape or weight interfered with your ability to concentrate on things?	Not at all/Yes occasionally/Yes most of the time
		Have you been afraid that you may become fat?	〃
		Have you felt fat?	〃
		Have you had a strong desire to lose weight?	〃
		Has your weight influenced how you think about yourself as a person?	〃
		Have you felt dissatisfied about your weight?	〃
		Have you felt dissatisfied about your shape?	〃
		Have you felt concerned about other people seeing you eat?	〃
		Have you felt uncomfortable seeing your body in a mirror?	〃
		Have you experienced a loss of control over eating?	〃
		Image perception score ^b^	10–30
**C (32 weeks)**	c330–c350	Have you been on a diet this pregnancy?	Yes/No
		Apart from this pregnancy have you ever gone on a diet to lose weight?	Yes/No
		If yes: How often? How long do your diets usually last?	1–2, 3–5/6–10/More than 10 times Under 1 month/1–3 months/More than 3 months
		Do you feel now you’ve put on too much weight?	Yes most of the time/Yes occasionally/No not at all
		Do you feel uncomfortable seeing your body in the mirror?	〃
		Have you had a strong desire to lose weight at any time during this pregnancy?	〃
		Do you feel dissatisfied about your shape?	〃
		Have you experienced any loss of control over eating during this pregnancy?	〃
		Are you concerned about losing any extra weight you’ve gained in this pregnancy?	〃

^a^b822–b832 variables are a repeat of this this question but pre-pregnancy. ^b^Sum of scores in previous question (Fairburn and Stein, Oxfordshire Eating Survey, 1990).

***Data available: Dietary variables collected in pregnancy (G0) available in the ALSPAC database***

- Frequency of weekly consumptions of 43 foods/food groups and eight daily foods/drinks from FFQ (c200 to c219, c222 to c247, c250).
- Nutrient intakes per day were estimated from the FFQ (excluding alcohol and dietary supplements) for: (1) energy, protein, carbohydrate, cholesterol, fat, saturated fat, monounsaturated fat, polyunsaturated fat, total sugar, non-milk extrinsic sugars (NMES), starch, non-starch polysaccharide (NSP), 12 vitamins and nine minerals (c3800 to c3805, c3809 to c3836; and (2) *n*-3 fatty acids, EPA and DHA from fish (c3806 to c3808). These are summarised in Table 6.
- Data from additional questions in FFQ comprising: How often food was fried (c220); Whether some or all of the fat was cut off meat (c221); Frequency of selection of low calorie/diet soft drinks (c247); Types of bread (c252 to c254); Frequency of bread/chapatis (c255); Frequency of take-away foods (c251); Types of fat used on bread, vegetables and for frying (c260 to c275); Type of milk used and frequency of use in tea/coffee, to drink, in cereal/puddings and in milky drinks (c276 to c289); Consumption of caffeinated/decaffeinated tea/coffee and use of sugar in tea/coffee (c300 to c309); Frequency of caffeinated/decaffeinated cola (c310 to c312); Frequency of herbal teas (c315, c316); Weekly caffeine intake (c317, c318); Frequency of purchase of types of organic foods and other health foods (c320 to c324); Whether self-identified as a vegan or vegetarian and if so for how long (c340 to c343).
- Dietary patterns (n=5) identified using principal components analysis (PCA) (excluding alcohol) (c3840 to c3844).
- Other drinks: tea and coffee (a210, a211, a220 to a231, b740 to b751); herbal tea (c315, c316); cola caffeinated/decaffeinated (a232 to a237, b761 to b766); milk (a270 to a272, b770 to b772); other (a273, a274, b773 to b775).
- Supplement and diet food intakes (b140 to b149, c110 to c115).
- Food-related behaviours: dieting during this pregnancy, body image, concern about weight gain, bingeing frequency (b800 to b832, c345 to c360).

**Table 6.** Descriptive statistics (mean and SD) for estimated daily nutrient intakes in mothers at 32 weeks of pregnancy calculated from the food frequency questionnaire.

Nutrient (per day)	Variable	ALSPAC G0 32 weeks pregnancy (n=11,978) ^a^
Energy (MJ)	c3804	7.23 (2.01)
Protein (g)	c3821	69.3 (19.7)
Total fat (g)	c3805	71.0 (23.4)
Saturated fat (g)	c3824	30.0 (11.5)
Monounsaturated fat (g)	c3813	24.0 (8.0)
Polyunsaturated fat (g)	c3819	12.3 (4.6)
Cholesterol (mg)	c3803	217 (89)
Carbohydrate (g)	c3801	213 (63)
Starch (g)	c3827	116 (36)
Sugar (g)	c3828	96 (39)
NMES (g)	c3816	61 (35)
NSP (g)	c3817	14.9 (5.1)
Calcium (mg)	c3800	938 (287)
Potassium (mg)	c3820	2879 (738)
Sodium (mg)	c3826	2193 (648)
Phosphorus (mg)	c3818	1247 (348)
Magnesium (mg)	c3812	247 (75)
Folate (μg)	c3809	243 (73)
Iron (mg)	c3811	10.2 (3.3)
Zinc (mg)	c3836	8.2 (2.4)
Vitamin C (mg)	c3831	79.5 (35.2)
Thiamin (mg)	c3829	1.43 (0.42)
Riboflavin (mg)	c3823	1.70 (0.56)
Retinol (μg)	c3822	367 (362)
Vitamin E (mg)	c3835	8.5 (4.1)
Vitamin D (μg)	c3834	3.8 (2.1)
Vitamin B _6_ (mg)	c3832	1.9 (0.5)
Vitamin B _12_ (μg)	c3833	4.9 (2.7)
Niacin equivalents (mg)	c3815	30.3 (9.0)
Niacin (mg)	c3814	15.9 (5.2)
Tryptophan (mg)	c3830	14.4 (4.1)
Carotene (μg)	c3802	2128 (1177)
Selenium (μg)	c3825	70.6 (27.9)
Iodine (μg)	c3810	148 (49)
Alcohol (units per week)	c373	1.7 (3.7)
*n*-3 Fatty acids from fish only (g)	c3806	0.15 (0.15)
DHA from fish only (g)	c3807	0.07 (0.07)
EPA from fish only (g)	c3808	0.05 (0.05)

^a^Mothers’ 32 weeks’ pregnancy FFQ nutrient data does not include intakes from alcohol or supplements NSP, non-starch polysaccharide; NMES, non-milk extrinsic sugars; DHA, docosahexaenoic acid; EPA, eicosapentaenoic acid

Data are also available on alcohol consumption (a213, a250 to a261, b754 to b769, c361 to c373). The dietary data available in ALSPAC including G0 have been reviewed by Emmett ^1^. Diet in pregnancy in ALSPAC has been fully described by Rogers *et al.* (1998) ^57^. Dietary patterns were defined and described by Northstone *et al.* ^19^

### Derivation of dietary variables

***1. Nutrient intakes***. The FFQ was used to calculate an approximate daily nutrient intake for each participant. Each food group was assigned a composition, based on consideration of how often various foods included in that food group were consumed, and using an amount equivalent to one portion of that food group. For example, the foods included in calculation of the nutrient content of one portion of ‘leafy green vegetables’ were 0.4 portions of cabbage, 0.4 portions of Brussel sprouts, and 0.2 portions of spring greens. Standard portions suitable for women were assumed throughout the questionnaire based on published national data ^58^. The foods included in the calculation of one portion of ‘other root vegetables (i.e. not carrots)’ were 0.4 portions of swede, 0.4 portions of parsnip and 0.2 portions of turnip. A list of the foods assigned to each question for use in calculating nutrient intakes is given in Table 7. When a question on food consumption had not been answered, it was assumed that the food was rarely or never eaten. Vitamins and minerals taken as supplements and alcohol intake were not included in the calculations (the questions about alcohol consumption were added one-third of the way through data collection so for many women the data are missing; the questions on supplements are not detailed enough to enable complete nutrient derivation).

**Table 7.** A list of the foods assigned to each question for use in calculating nutrient intakes.

Food code ^a^	Name	Weight (g)
	**Question c1a**	
504	Beefburgers, chilled/frozen, fried	40
529	Pork sausages, chilled, grilled	40
	**Question c1b**	
508	Cornish pasty	39
520	Pork pie, individual	35
522	Sausage rolls, flaky pastry, homemade	15
534	Steak and kidney/Beef pie, individual, chilled/frozen, baked	40
	**Question c1b (used for vitamin E and retinol)**	
508	Cornish pasty	39
520	Pork pie, individual	35
522	Sausage rolls, flaky pastry, homemade	15
535	Steak and kidney pie, single crust, homemade	30
	**Question c1c Eats all fat on meat**	
386	Beef, topside, roasted well-done, lean and fat	23
412	Lamb, shoulder, whole, roasted, lean and fat	23
420	Pork, loin chops, grilled, lean and fat	30
358	Bacon rashers, middle, grilled, lean and fat	12
	**Question c1c Doesn’t eat fat on meat**	
387	Beef, topside, roasted well-done, lean	23
413	Lamb, shoulder, whole, roasted, lean	23
422	Pork, loin chops, grilled, lean	30
356	Bacon rashers, lean only, grilled, average	12
	**Question c1c Eats some of fat on meat**	
387	Beef, topside, roasted well-done, lean	11
386	Beef, topside, roasted well-done, lean and fat	11
413	Lamb, shoulder, whole, roasted, lean	11
412	Lamb, shoulder, whole, roasted, lean and fat	11
422	Pork, loin chops, grilled, lean	15
420	Pork, loin chops, grilled, lean and fat	15
356	Bacon rashers, lean only, grilled, average	6
358	Bacon rashers, lean and fat, grilled, middle	6
	**Question c1d Eats all fat on meat**	
439	Chicken, roast, meat and skin	100
	**Question c1d Doesn’t eat fat on meat**	
438	Chicken, roast, meat only	100
	**Question c1d Eats some of fat on meat**	
438	Chicken, roast, meat only	50
439	Chicken, roast, meat and skin	50
	**Question c1e**	
474	Heart, ox, stewed	25
482	Kidney, pig, stewed	28
488	Liver, lamb, fried	25
517	Pate, liver	20
	**Question c1e (used for vitamin C and vitamin E)**	
474	Heart, ox, stewed	33
482	Kidney, pig, stewed	37
488	Liver, lamb, fried	33
	**Question c1f**	
564	Cod, baked, fillets	30
580	Haddock, coated in crumbs, fried in blended oil	30
597	Plaice, in batter, fried in blended oil	50
653	Fish fingers, grilled	20
	**Question c1f (used for folate and pyridoxine)**	
564	Cod, baked, fillets	30
16049	Haddock, steamed	30
16110	Plaice, in batter, fried in blended oil	50
653	Fish fingers, grilled	20
	**Question c1f (used for vitamin E)**	
564	Cod, baked, fillets	40
16056	Haddock, in flour, fried in blended oil	40
16114	Plaice, in crumbs, fried in blended oil	50
	**Question c1g**	
613	Herring, grilled	20
615	Kipper, baked	22
618	Mackerel, fried	27
621	Pilchards, canned in tomato sauce	18
625	Salmon, canned	17
632	Tuna, canned in brine, drained	15
	**Question c1g (used for folate, zinc and vitamin E)**	
613	Herring, grilled	24
615	Kipper, baked	26
16198	Mackerel, canned in tomato sauce	20
625	Salmon, canned	20
632	Tuna, canned in brine, drained	18
	**Question c1g (used for retinol)**	
613	Herring, grilled	20
615	Kipper, baked	22
618	Mackerel, fried	27
621	Pilchards, canned in tomato sauce	18
625	Salmon, canned	17
16228	Tuna, raw	15
	**Question c1h**	
636	Crab, canned in brine, drained	21
639	Prawns, boiled	15
641	Scampi, in breadcrumbs, frozen, fried	43
645	Mussels, boiled	10
	**Question c1h (used for folate and pyridoxine)**	
16232	Crab, boiled	28
16236	Lobster, boiled	28
16256	Mussels, boiled	13
	**Question c1h (used for vitamin E)**	
16236	Lobster, boiled	21
16238	Prawns, raw	15
16256	Mussels, boiled	10
16265	Squid, in batter, fried in blended oil	30
	**Question c1h (used for retinol)**	
16234	Crab, canned in brine, drained	21
16239	Prawns, boiled	15
16243	Scampi, in breadcrumbs, frozen, fried in blended oil	43
16252	Cockles, boiled	10
	**Question c1i**	
293	Eggs, chicken, boiled	17
301	Omelette, plain	40
303	Quiche, cheese and egg	47
	**Question c1j**	
228	Cheddar, average	23
237	Edam	23
	**Questions c1k**	
170	Pizza, cheese and tomato	115
15254	Pizza, cheese and tomato, retail, frozen	115
	**Questions c1k (used for carotene, vitamin C and retinol)**	
170	Pizza, cheese and tomato	230
	**Question c1l**	
679	Chips, retail, fried in blended oil	42
681	Chips, straight cut, frozen, fried in blended oil	33
680	Chips, French fries, retail	23
687	Oven chips, frozen, baked	33
674	Chips, homemade, fried in blended oil	33
	**Question c1l (used for folate)**	
681	Chips, straight cut, frozen, fried in blended oil	55
687	Oven chips, frozen, baked	55
674	Chips, homemade, fried in blended oil	55
	**Question c1l (used for vitamin E)**	
13022	Chips, retail, fried in vegetable oil	70
13023	Chips, French fries, retail	39
13029	Oven chips, frozen, baked	55
	**Question c1m**	
671	Old potatoes, roast in blended oil	103
	**Question c1m**	
673	Old potatoes, roast in lard	103
	**Question c1m**	
672	Old potatoes, roast in corn oil	103
	**Question c1n**	
661	New potatoes, boiled in unsalted water	20
665	Old potatoes, baked, flesh and skin	30
666	Old potatoes, baked, flesh only	27
668	Old potatoes, boiled in unsalted water	60
	**Question c1o**	
19	Brown rice, boiled	27
23	White rice, easy cook, boiled	108
	**Question c1o (used for pyridoxine)**	
23	White rice, easy cook, boiled	135
	**Question c1p**	
30	Spaghetti, white, boiled	120
32	Spaghetti, wholemeal, boiled	30
	**Question c1q**	
1037	Potato crisps	27
	**Question c3a**	
694	Baked beans, canned in tomato sauce, re-heated	135
	**Question c3b**	
732	Peas, frozen, boiled in unsalted water	35
733	Peas, canned, re-heated, drained	18
824	Sweetcorn, kernels, canned, re-heated, drained	21
	**Question c3c**	
747	Brussels sprouts, boiled in unsalted water	36
750	Cabbage, boiled in unsalted water, average	38
817	Spring greens, boiled in unsalted water	19
	**Question c3c (use for vitamin E)**	
747	Brussels sprouts, boiled in unsalted water	36
750	Cabbage, boiled in unsalted water, average	38
13345	Spinach, boiled in unsalted water	18
	**Question c3d**	
13083	Green beans/French beans, boiled in unsalted water	23
13172	Broccoli, green, boiled in unsalted water	21
13217	Cauliflower, boiled in unsalted water	23
13265	Leeks, boiled in unsalted water	19
	**Question c3e**	
755	Carrots, old, boiled in unsalted water	60
	**Question c3f**	
800	Parsnip, boiled in unsalted water	26
820	Swede, boiled in unsalted water	24
834	Turnip, boiled in unsalted water	12
	**Question c3g**	
767	Cucumber, raw	23
777	Lettuce, average, raw	30
827	Tomatoes, raw	60
	**Question c3h**	
856	Apples, eating, average, raw	17
867	Bananas	17
903	Grapes, average	17
931	Oranges	21
938	Peaches, raw	18
942	Pears, average, raw	28
	**Question c3h (used for vitamin E)**	
856	Apples, eating, average, raw	17
867	Bananas	17
903	Grapes, average	17
931	Oranges	21
942	Pears, average, raw	28
14220	Plums, Victoria, raw	18
	**Question c3i**	
832	Tomatoes, canned, whole contents	80
4-885	Orange juice, unsweetened	80
	**Question c3i (used for vitamin E)**	
832	Tomatoes, canned, whole contents	80
1091	Orange juice, unsweetened	80
	**Question c3j**	
1087	Apple juice, unsweetened	32
1091	Orange juice, unsweetened	96
1092	Pineapple juice, unsweetened	32
	**Question c3k**	
117	Gateau	14
154	Crumble, fruit	28
157	Fruit pie, pastry top and bottom	18
164	Sponge pudding	18
274	Cheesecake, frozen	20
287	Rice pudding, canned	25
	**Question c3k (used for carotene)**	
117	Gateau	14
154	Crumble, fruit	28
157	Fruit pie, pastry top and bottom	18
164	Sponge pudding	18
283	Milk pudding, made with whole milk	25
285	Mousse, chocolate	10
	**Question c3k (used for folate)**	
117	Gateau	14
154	Crumble, fruit	28
157	Fruit pie, pastry top and bottom	18
164	Sponge pudding	18
274	Cheesecake, frozen	20
283	Milk pudding, made with whole milk	25
	**Question c3l**	
73	Muesli, Swiss style	17
76	Porridge, made with water	53
80	Ready Brek	10
	**Question c3m**	
65	All-Bran	10
66	Bran Flakes	8
72	Fruit 'n Fibre	8
90	Weetabix	10
	**Question c3m (used for vitamin C)**	
65	All-Bran	13
66	Bran Flakes	10
90	Weetabix	13
	**Question c3m (used for vitamin E)**	
65	All-Bran	13
83	Shredded Wheat	15
90	Weetabix	13
	**Question c3n**	
69	Corn Flakes	6
71	Frosties	6
81	Rice Krispies	6
84	Shreddies	9
86	Special K	6
	**Question c3n (use for vitamin E)**	
69	Corn Flakes	8
81	Rice Krispies	8
86	Special K	8
88	Sugar Puffs	8
	**Question c3o**	
112	Fancy iced cakes, individual	6
114	Fruit cake, rich	14
121	Sponge cake, jam filled	12
136	Doughnuts, jam	15
138	Eccles cake	9
	**Question c3o (use for folate and vitamin E)**	
98	Flapjacks	12
114	Fruit cake, rich	14
120	Sponge cake, fatless	12
136	Doughnuts, jam	15
138	Eccles cake	9
	**Question c3o (use for carotene and retinol)**	
98	Flapjacks	15
114	Fruit cake, rich	18
120	Sponge cake, fatless	15
138	Eccles cake	11
	**Question c3p**	
95	Crispbread, rye	20
	**Question c3q**	
97	Digestive biscuits, plain	3
104	Semi-sweet biscuits	2
105	Short-sweet biscuits	3
1020	KitKat	6
	**Question c3q (use for folate)**	
97	Digestive biscuits, plain	3
104	Semi-sweet biscuits	2
105	Short-sweet biscuits	3
106	Shortbread	3
	**Question c3q (use for vitamin E)**	
96	Digestive biscuits, chocolate	3
104	Semi-sweet biscuits	2
105	Short-sweet biscuits	3
1020	KitKat	6
	**Question c3r**	
1021	Mars bar	33
1024	Twix	28
	**Question c3r (use for folate and zinc)**	
17084	Chocolate-covered bar with fruit/nut wafer/biscuit	19
17095	Milky Way	9
17097	Snickers	20
	**Question c3r (use for vitamin E)**	
1024	Twix	19
1019	Crème Eggs	13
17084	Chocolate covered bar with fruit/nut wafer/biscuit	19
	**Question c3s**	
705	Chickpeas, whole, dried, boiled in unsalted water	40
713	Lentils, red, split, dried, boiled in unsalted water	40
718	Red kidney beans, canned, re-heated, drained	40
	**Question c3s (use for total fat and energy)**	
15132	Curry, mung bean dahl and tomato	50
15103	Curry, chickpea, whole, basic	50
15120	Curry, lentil, red/masoor dahl, punjabi	50
	**Question c3s (use for vitamin E)**	
703	Butter beans, canned, re-heated, drained	40
705	Chickpeas, canned, re-heated, drained	40
718	Red kidney beans, canned, re-heated, drained	40
	**Question c3t**	
972	Almonds	8
974	Brazil nuts	8
980	Hazelnuts	8
990	Peanuts, roasted and salted	28
	**Question c3u**	
724	Tofu, soya bean, steamed, fried	80
	**Question c3u (used for fatty acids)**	
15319	Tofu burger	90
15320	Tofu spread	30
	**Question c3u (used for vitamin E)**	
13119	Tofu, soya bean, steamed	80
15319	Tofu burger, baked	90
	**Question c3u (used for total fat and energy)**	
724	Tofu, soya bean, steamed, fried	80
15319	Tofu burger, baked	90
15320	Tofu spread	30
	**Question c3v**	
13088	Hummus	10
14847	Tahini paste	10
	**Question c3v (used for fatty acids)**	
14847	Tahini paste	20
	**Question c3w**	
15326	Vegeburger mix, made up with water, fried in vegetable oil	37
15331	Vegeburger, retail, grilled	47
2117	Soya mince, made up with water	36
	**Question c3x**	
17088	Chocolate, fancy and filled	10
17089	Chocolate, milk	30
17090	Chocolate, plain	10
	**Question c3y**	
17104	Chew sweets	6
17108	Fruit pastilles	7
17109	Fudge	5
17112	Liquorice allsorts	9
17117	Peppermints	5
17120	Toffees, mixed	8
	**Question c3y (used for zinc)**	
17104	Chew sweets	7
17108	Fruit pastilles	8
17109	Fudge	6
17112	Liquorice allsorts	11
17120	Toffees, mixed	9
	**Question c3y (used for niacin)**	
17108	Fruit pastilles	8
17109	Fudge	6
17112	Liquorice allsorts	11
17117	Peppermints	6
17120	Toffees, mixed	9
	**Question c3y (used for vitamin E)**	
17104	Chew sweets	7
17108	Fruit pastilles	8
17109	Fudge	6
17112	Liquorice allsorts	11
17117	Peppermints	6
	**Question c4**	
17175	Cola	110
17177	Fruit juice drink, carbonated, ready to drink	110
17179	Lemonade	110
	**Question c7a**	
49	White bread, sliced	36
	**Question c7b**	
33	Brown bread, average	18
39	Granary bread	18
	**Question c7c**	
56	Wholemeal bread, average	36
	**Question c7d**	
36	Chapatis, made without fat	28
43	Naan bread	40
	**Question c7d (used for fatty acids)**	
17009	Ghee, vegetable	15
	**Question c8a (used for bread)**	
306	Butter	100
	**Question c8a (used for cooking fat)**	
316	Compound cooking fat	10
317	Dripping, beef	10
335	Ghee, butter	5
336	Ghee, palm	5
318	Lard	35
306	Butter	35
	**Question c8a cooking fat (used for carotene and retinol)**	
17004	Compound cooking fat	10
17007	Ghee, butter	5
17008	Ghee, palm	5
17010	Lard	45
17013	Butter	35
	**Question c8b**	
310	Margarine, hard, animal and vegetable fat	25
311	Margarine, hard, vegetable fat only	25
312	Margarine, soft, animal and vegetable fat	25
313	Margarine, soft, vegetable fat only	25
	**Question c8b (used for vitamin E)**	
17018	Margarine, hard, animal and vegetable fats	34
17020	Margarine, soft, not polyunsaturated	33
17021	Margarine, soft, polyunsaturated	33
	**Question c8c**	
314	Fat spread (70% fat), polyunsaturated	100
	**Question c8c (used for vitamin E)**	
17023	Fat spread (70% fat) polyunsaturated fat	100
	**Question c8d**	
308	Low fat spread	100
	**Question c8e**	
322	Corn oil	30
324	Olive oil	10
331	Soya oil	30
332	Sunflower oil	30
	**Question c8f**	
333	Vegetable oil, blended, average	100
	**Question c10a**	
190	Whole milk, pasteurised	100
	**Question c10b**	
186	Semi-skimmed milk, pasteurised	100
	**Question c10c**	
182	Skimmed milk, pasteurised	100
	**Question c10d**	
193	Whole milk, sterilised	100
	**Question c10f**	
204	Goats’ milk, pasteurised	50
208	Sheep milk, raw	50
	**Question c10g**	
209	Soya milk, plain	100

^a^Food codes: 5th edition of *McCance and Widdowson’s The composition of foods* and its supplements ^59– 64^.

The approximate weekly intake was then calculated by multiplying the weekly frequency of consumption of a food/food group by the nutrient content (obtained from the 5th edition of *McCance and Widdowson’s The Composition of Foods* and its supplements ^59– 64^) of a standard portion ^58^ of that food, and summing this for all the foods consumed. The weekly frequencies of consumption assumed for each of the options ticked in the questionnaire were as follows: ‘Never or rarely’ = 0, ‘Once in 2 weeks’ = 0.5, ‘1–3 times per week’ = 2, ‘4–7 times per week’ = 5.5, and ‘More than once a day’ = 10. The question on bread consumption asked how many pieces of bread, rolls or chapatis were eaten on a normal day: the frequency options were <1, 1–2, 3–4 and ≥5. Total milk consumption was calculated by summing the likely amount of milk drunk in tea and coffee, on breakfast cereals, as milk on its own and in flavoured milk drinks (the data in the additional questions were used in this process).

*n*-3 Fatty acids from fish were calculated from the three questions about seafood consumption: ‘How many times nowadays do you eat: (a) white fish (cod, haddock, plaice, fish fingers, etc.); (b) dark or oily fish (tuna, sardines, pilchards, mackerel, herring, kippers, trout, salmon, etc.); (c) shellfish (prawns, crabs, cockles, mussels etc.)?’. Portion sizes were based on typical consumption patterns in the UK. Fatty acid compositions were based on profiles of typical British species ^23, 65^. The subtypes of fish used to code white fish, other fish and shellfish are shown in Table 8.

**Table 8.** Subtypes of fish used to code white fish, other fish and shellfish.

White fish	50 g fried plaice in batter + 30 g baked cod fillets + 30 g fried haddock in crumbs + 20 g grilled fish fingers
Other fish	60 g tuna canned in brine + 12 g homemade salmon fish cakes + 10 g canned pink salmon + 8 g brown trout + 5 g steamed salmon + 5 g sardines canned in oil + 5 g pilchards canned in tomato sauce + 3 g sardines canned in tomato sauce
Shellfish	43 g scampi, breadcrumbed and fried + 21 g canned crab + 10 g boiled mussels + 15 g boiled prawns

Estimates of the *n*-3 fatty acid intake for each portion of white fish were 0.32 g, oily fish 0.89 g and shellfish 0.35g ^65^. Eicosapentaenoic acid (EPA) and docosahexaenoic acid (DHA) from fish were calculated using the same method.

Caffeine intake per week was computed from the questions on tea, coffee and cola, after taking account of decaffeinated drinks. It was assumed that there were 27 mg per cup of tea, 57 mg per cup of coffee and 20 mg per drink of cola.

Syntax to derive each nutrient was written and run in SPSS version 21 ^66^. If the estimated intakes resulted in unrealistically high or low values, those individuals were removed from the estimated intakes. The cut-off levels are shown in Table 9. This led to the exclusion of 422 participants for the nutrients and 413 participants for the *n*-3 fatty acids. The nutrient values obtained were then divided by seven to convert to a daily intake. Derived nutrient data currently available in ALSPAC are based on version 6 of syntax (year 2012); previous publications are based on earlier versions, e.g. Rogers & Emmett ^57^ (1998) uses version 1 of the 32-week pregnancy data (see Table 10).

**Table 9.** Parameters for removal of individuals with unrealistically high or low nutrient intake values.

Energy	SELECT IF ((Wenergy > 15000) & (Wenergy < 120000))
Calcium	SELECT IF(Ccalcium < 2500)
Carbohydrate	SELECT IF(Ccarbohydrate < 600)
Carotene	SELECT IF(Ccarotene < 8000)
Cholesterol	SELECT IF(Ccholesterol < 1000)
Folate	SELECT IF(Cfolate < 700)
Iodine	SELECT IF(Ciodine < 450)
Iron	SELECT IF(Ciron < 40)
Magnesium	SELECT IF(Cmg < 700)
MUFA	SELECT IF(Cmono < 70)
Niacin equivalents	SELECT IF(Cnceq < 90)
NMES	SELECT IF(Cnmesugars < 350)
Phosphorus	SELECT IF(Cphos < 3500)
Protein	SELECT IF(Cprotein < 200)
Retinol	SELECT IF(Cretinol < 8000)
Riboflavin	SELECT IF(Cribo < 7)
Selenium	SELECT IF(Cselenium < 350)
Starch	SELECT IF(Cstarch < 400)

MUFA, monounsaturated fatty acids; NMES, non-milk extrinsic sugars.

**Table 10.** Data analysis versions.

The publication describing the pregnancy diet uses version 1 of the data ^57^. Version 6, which is the data version available from ALSPAC in 2024, builds on version 5, which included changes to the way milk, bread, spreading and cooking fats were calculated to incorporate responses in which participants ticked more than one type or ‘Sometimes’ rather than ‘Usually’ in the questionnaire. These changes made little difference to overall mean intakes between version 4 and 5 but affected about one-third of individual intakes particularly of fat fractions (polyunsaturated and monounsaturated fats). Some nutrients such as vitamin B _12_ were very little affected. Some changes were made to the calculation of NMES including the addition of sugar from fruit juice: this meant that it is equivalent to free sugars. In version 6 the missing data for slices of bread per day were recoded to 1.5 (average amount) and for number of slices spread with fat – missing goes to 2 and 7–14 times a day were recoded to allow a maximum of 6. Soft drinks did not have a frequency in this questionnaire so the most common frequency in the women’s 47-month (J) FFQ was used (2/week). A new file with weight of food for each food group was made. The original versions (1, 2 and 3) of the pregnancy nutrients used a different database (from Microdiet) and did not include all nutrients required; versions 4 and 5 used a database contemporary to the time of data collection (5th edition of *McCance and Widdowson’s The composition of foods* and its supplements ^59– 64^.

***2. Dietary patterns.*** The derivation of dietary patterns by PCA has been described by Northstone *et al.* ^40^. Five dietary patterns were identified and named: ‘Health conscious’ (high positive loadings on salad, fruit, rice, pasta, breakfast cereal, fish, eggs, pulses, fruit juices, white meat, non-white bread), ‘Traditional’ (high positive loadings on vegetables, red meat and poultry), ‘Processed’ (high positive loadings on high-fat processed foods), ‘Confectionary’ (high positive loadings on snack foods with high sugar content) and ‘Vegetarian’ (high positive loadings on meat substitutes, pulses, nuts and herbal tea and high negative loadings on red meat and poultry). Scores for each dietary pattern for each participant are available (c3840 to c3844).

### Examples of additional variables that can be derived by researchers

Derived variables such as dietary style, diet quality scores and dietary variety scores can be calculated by researchers from the variables available in the dataset (see Table 2). AOAC fibre can be derived from NSP fibre (AOAC (g) = NSP (g) × 1.33). The NMES variable in version 6 is equivalent to free sugars. Daily consumption of 43 food items from the weekly FFQ questions and five foods eaten daily from the additional question can be used to derive the daily weight (g/day) consumed or specific contributions to the intakes of individual nutrients. These foods can be grouped, as desired, to answer specific research questions.

## Dataset validation

### Validation

The FFQ was not validated prior to use. However, the questions about fish consumption were shown to be associated with both *n*-3 long-chain PUFA in maternal serum ^23, 67^ and mercury concentrations in maternal blood ^33^. Oily fish consumption was validated by comparison with the erythrocyte fatty acid composition of blood samples obtained during pregnancy ^57^.

### Misreporting

Misreporting of dietary data is a common problem in dietary assessment and can lead to under- or over-estimates of nutrient intakes ^68^. This can arise from misunderstanding the questions, missing out questions, an inability to select an appropriate response or altering reporting to make it more ‘acceptable’. There are several methods available to researchers to address misreporting, including individualised methods based on predicted energy requirement that take into account age, sex and body weight allowing for a standard level of physical activity ^69^. For these pregnancy FFQ data it would be possible to use pre-pregnancy weight as self-reported by the woman (dw002). Identification of misreporters can be used to exclude participants from analyses and/or conduct sensitivity analyses (e.g. Jones *et al.* ^70^), or can be used as a confounder (e.g. Buckland *et al.* ^71^).

### Standardisation

Nutrient and food group intakes can be standardised for energy intake to enable interpretation of data. Most simply this can be done by calculating the intake as a percentage of energy or per unit of energy. Variables can also be standardised for body weight if preferred (for the pregnancy FFQ data it would be possible to use pre-pregnancy weight as self-reported by the woman or weights obtained in the antenatal clinic as noted earlier).

## Abbreviations

ALSPAC, Avon Longitudinal Study of Parents and Children; G0, Generation 0 (ALSPAC mothers); NMES, non-milk extrinsic sugars; NSP, non-starch polysaccharide; FFQ, food frequency questionnaire.

## Ethics and consent statement

Ethics approval for the study was obtained from the ALSPAC Law and Ethics committee and local research ethics committees. Implied consent for the use of data collected via questionnaires and informed consent for information acquired for tests in clinics were obtained from participants following the recommendations of the ALSPAC Ethics and Law Committee at the time ^52^ (Bristol and Weston Health Authority: E1808 Children of the Nineties: Avon Longitudinal Study of Pregnancy and Childhood (ALSPAC) (28 November 1989); Southmead Health Authority: 49/89 Children of the Nineties – ‘ALSPAC’ (5 April 1990); Frenchay Health Authority: 90/8 Children of the Nineties (28 June 1990)). Study participants have the right to withdraw their consent for elements of the study or from the study entirely at any time. Full details for the ALSPAC consent procedures are available on the study website ^53^.

## Data Availability

The ALSPAC study website contains details of all the data that are available through a fully searchable data dictionary and variable search tool: http://www.bris.ac.uk/alspac/researchers/data-access/data-dictionary/. ALSPAC data access is through a system of managed open access. The steps below highlight how to apply for access to all ALSPAC data including those described in this data note: Please read the ALSPAC access policy ( http://www.bristol.ac.uk/media-library/sites/alspac/documents/researchers/data-access/ALSPAC_Access_Policy.pdf) which describes the process of accessing the data and samples in detail, and outlines the costs associated with doing so. You may also find it useful to browse our fully searchable research proposals database ( https://proposals.epi.bristol.ac.uk/?q=proposalSummaries), which lists all research projects that have been approved since April 2011. Please submit your research proposal ( https://proposals.epi.bristol.ac.uk/) for consideration by the ALSPAC Executive Committee. You will receive a response within 10 working days to advise you whether your proposal has been approved. ALSPAC questionnaires and details of clinical measures are available on the ALSPAC website ^72^. This project used the following questionnaires ^73^: Questionnaire A Your Environment Questionnaire B Having a Baby Questionnaire C Your pregnancy
