# Supplementary material for: Dietary and related data collected during pregnancy in the Avon Longitudinal Study of Parents and Children (ALSPAC)
Source: Wellcome Open Res. 2025 Feb 24;10:6. Originally published 2025 Jan 14. [Version 2] doi: 10.12688/wellcomeopenres.23464.2 (PMC11904399; doi:10.12688/wellcomeopenres.23464.2)
Supplement: Peer review history

# Reviewer report: Reviewer response for version 2

**DOI:** 10.21956/wellcomeopenres.26291.r120083

**Published:** 2025-03-24

**Authors:** Charles Businge

**Affiliations:** 1 Walter Sisulu University, Mthatha, South Africa

***Dietary and related data collected during pregnancy in the Avon Longitudinal Study of Parents and Children (ALSPAC)***

 This is a valuable manuscript in which the authors explain the rationale of their project, precisely the type of nutritional data they are making available for secondary analysis, the methods used during data collection, and how they can be accessed and used.

**The abstract**

 In the abstract, the authors have provided a brief history of the cohort, a concise summary of the type of data they are making available, the frequency, gestation age, and strategies used for data collection, and how it can be linked to related social demographic data sets to test hypotheses.

**Introduction**

 The authors have explained the importance of the data set to the broader scientific community. They also articulated the importance of explicitly defining the methods used to assemble the data to guide and enable proper interpretation and further analysis by potential users.

**Methods**

 The authors gave an elaborate description of the processes of data collection at various gestational ages, how dietary variables were derived, and how they used the principal component analysis to identify the five major dietary patterns of the members of the cohort. They have also proposed methods potential users could employ to derive more variables. Furthermore, they have provided links and clear steps that may be used to access this rich dataset.

 The authors carried out a post-hoc validation of their food frequency questionnaire. It is not explicit why they were not able to validate the FFQ before the data collection.

 The authors have generously proposed ways to identify misreporting, which can be a source of bias. It is interesting to know if they applied some of these methods to clean the dataset or to flag participants with misreported data.

Are sufficient details of methods and materials provided to allow replication by others?

Yes

Is the rationale for creating the dataset(s) clearly described?

Yes

Are the datasets clearly presented in a useable and accessible format?

Yes

Are the protocols appropriate and is the work technically sound?

Yes

Reviewer Expertise:

Obstetrics and Gynaecology, micronutrients

I confirm that I have read this submission and believe that I have an appropriate level of expertise to confirm that it is of an acceptable scientific standard.

# Reviewer report: Reviewer response for version 2

**DOI:** 10.21956/wellcomeopenres.26291.r119397

**Published:** 2025-03-12

**Authors:** Stephanie Atkinson

**Affiliations:** 1 McMaster University, Ontario, Canada

I was able to access the authors' comments in the link that was sent yesterday.  They have not made any changes of significance. I understand their rationale for not including the exact costs in the article. In my experience, people wanting to access such data often do not realize the extent of the costs involved, hence why I was suggesting that information on costs be included. The authors state that cost information is available in the online documents for which links are provided, so I guess that is satisfactory.

Are sufficient details of methods and materials provided to allow replication by others?

Yes

Is the rationale for creating the dataset(s) clearly described?

No

Are the datasets clearly presented in a useable and accessible format?

Yes

Are the protocols appropriate and is the work technically sound?

Yes

Reviewer Expertise:

DOHaD research using both epidemiological and randomized trial study designs

I confirm that I have read this submission and believe that I have an appropriate level of expertise to confirm that it is of an acceptable scientific standard.

# Reviewer report: Reviewer response for version 1

**DOI:** 10.21956/wellcomeopenres.25874.r117190

**Published:** 2025-02-10

**Authors:** Stephanie Atkinson

**Affiliations:** 1 McMaster University, Ontario, Canada

This paper, which is categorized by Wellcome as a DATA NOTE, is a description of the ALSPAC longitudinal study which is well-known worldwide especially among researchers in the DOHaD field. It is generous of the study investigators to offer the insights into the current status of the study, and details of many aspects of the methodology used to conduct the study with an emphasis on collection, analysis and interpretation of dietary aspects including the actual questionnaires used. 

- ** Clarity of presentation**

The details of the study design and methodologies are well described and supported by supplementary materials many of which are offered via web links.  

 At the end of the paper there is a section on Data Availability that includes a link to a Data Access Policy. The summary in this document states: *“The purpose of this document is to describe in detail the general processes and procedures involved in accessing the ALSPAC resource (defined as both the data already collected and the participants themselves for the purposes of new data collection). We encourage and facilitate data sharing with all ‘bona fide’ researchers.”*

 The information in the Data Access Policy is similar to what is contained in the paper. It would be informative to provide some details of costs for accessing either the materials on methods or individual participant data.

- **Study design**

The description of how the ALSPAC study was conducted and follow-up measures are adequately described.

- **Methods and analysis**

The details of the collection of dietary data, its analysis, interpretation and limitations is extremely well done and would be very valuable to anyone setting up a study that included collection of dietary patterns or nutrient intakes. Details of methods used and links to access to all questionnaires are provided.

- **Statistical analysis and its interpretation  **

Statistical analysis was not included in the paper and was not really relevant although guidance on approaches to analysis might be informative.

- **Source data**  

Based on the  link to the Data Access Policy, it is assumed that ‘bona fide researchers’ can obtain access to the individualized data from the ALSPAC study. But as noted above, more details about this aspect would he informative.

Are sufficient details of methods and materials provided to allow replication by others?

Yes

Is the rationale for creating the dataset(s) clearly described?

No

Are the datasets clearly presented in a useable and accessible format?

Yes

Are the protocols appropriate and is the work technically sound?

Yes

Reviewer Expertise:

DOHaD research using both epidemiological and randomized trial study designs

I confirm that I have read this submission and believe that I have an appropriate level of expertise to confirm that it is of an acceptable scientific standard, however I have significant reservations, as outlined above.

# Response

**Published:** 2025-02-20

**Authors:** Caroline Taylor

**Affiliations:** Bristol Medical School, University of Bristol, Bristol, UK

Thank you for your review. In response to your specific comments:  

 1. **Clarity of presentation Costings: **The details of costings to access ALSPAC data are referred to in the 'Data availability' section, where we provide a link to the ALSPAC access policy, which includes costings. We prefer to not to include the precise costings in the data note as they are subject to change and may be out of date when the text is accessed in the future. No changes made.

 2. **Statistical analysis and interpretation:** We agree that it is not relevant for us to include details of statistical analyses here, as they will be determined by research groups depending on their research question(s). We have included information and references that give details of statistical methods for how data including dietary patterns have been derived. No changes made.  

 3. **Source data: **We have included details of a three-step process for researchers to gain access to ALSPAC data through a controlled access procedure overseen by the ALSPAC Executive Committee. This includes web links to the access policy, costs, research proposals database and proposal application site. No changes made.  

 4. Rationale for creating the dataset We have included detailed information on the rationale and methods, with appropriate citations, in the Methods section of the data note. If there is any specific information that has been identified as missing, we would be happy to add it.  No changes made at this stage.  

 5. Additional change made to add additional funder for JG.

# Reviewer report: Reviewer response for version 1

**DOI:** 10.21956/wellcomeopenres.25874.r117197

**Published:** 2025-02-06

**Authors:** Corinna Walsh

**Affiliations:** 1 University of the Free State, Bloemfontein, Free State, South Africa

This paper describes the methodology that was applied to assess dietary intake in participants of the Avon Longitudinal Study of Parents and Children (ALSPAC), a longitudinal birth cohort study based in the south-west of the UK. A major strength is very large sample size and related information that was collected.

 Limitations include the fact that questionnaires were self-administered and that quantitative data was not collected (portion sizes were estimated based on portions identified in other studies). It is uncertain whether these foods and portion sizes are valid for participants from immigrant groups. 

 Since this paper described the methodology used to collect dietary data, access to the datasets is not relevant.

 I have made additional comments on the PDF version of the paper.

Are sufficient details of methods and materials provided to allow replication by others?

Yes

Is the rationale for creating the dataset(s) clearly described?

Yes

Are the datasets clearly presented in a useable and accessible format?

No

Are the protocols appropriate and is the work technically sound?

Yes

Reviewer Expertise:

Nutrition and dietetics

I confirm that I have read this submission and believe that I have an appropriate level of expertise to confirm that it is of an acceptable scientific standard.
